# Quantitative interpretation explains machine learning models for chemical reaction prediction and uncovers bias

**DOI:** 10.1038/s41467-021-21895-w

**Published:** 2021-03-16

**Authors:** Dávid Péter Kovács, William McCorkindale, Alpha A. Lee

**Affiliations:** Cavendish Laboratory, University of Cambridge, Cambridge, UK

**Keywords:** Automation, Cheminformatics, Computational chemistry, Computational science

## Abstract

Organic synthesis remains a major challenge in drug discovery. Although a plethora of machine learning models have been proposed as solutions in the literature, they suffer from being opaque black-boxes. It is neither clear if the models are making correct predictions because they inferred the salient chemistry, nor is it clear which training data they are relying on to reach a prediction. This opaqueness hinders both model developers and users. In this paper, we quantitatively interpret the Molecular Transformer, the state-of-the-art model for reaction prediction. We develop a framework to attribute predicted reaction outcomes both to specific parts of reactants, and to reactions in the training set. Furthermore, we demonstrate how to retrieve evidence for predicted reaction outcomes, and understand counterintuitive predictions by scrutinising the data. Additionally, we identify Clever Hans predictions where the correct prediction is reached for the wrong reason due to dataset bias. We present a new debiased dataset that provides a more realistic assessment of model performance, which we propose as the new standard benchmark for comparing reaction prediction models.

## Abstract (web summary)

Machine learning algorithms offer new possibilities for automating reaction procedures. The present paper investigates automated reaction’s prediction with Molecular Transformer, the state-of-the-art model for reaction prediction, proposing a new debiased dataset for a realistic assessment of the model’s performance.

## Introduction

Organic synthesis remains a challenge in small molecule drug design, sinking time in the design-make-test cycle and potentially limiting the complexity of chemical space being explored^1,2^. The challenge of synthesis planning lies in searching through myriad of possible reactions to find optimal routes, and in predicting whether each possible reaction is indeed feasible and high yielding for the particular substrate in question. The problem of efficient search in synthesis has been recently addressed, inspired by innovations in computer science on searching and gameplay^3–7^. However, accurately predicting the outcome of chemical reactions remains a hurdle^8–10^.

The current state-of-the-art in reaction prediction is the Molecular Transformer^11^, which employs the transformer neural network architecture that was first introduced for neural machine translation^12^. The input to the model is a text representation of the chemical structures of the reactant and reagent, and the model performs machine translation to predict the most likely output molecule with a probability score. The Molecular Transformer achieves a 90% Top-1 accuracy on the USPTO dataset of organic reactions that were text mined from US patents^13^ and filtered^14^. Recent work shows that thorough dataset augmentation improves model performance by allowing it to consider different equivalent SMILES representations^15^.

However, a key stumbling block in the Molecular Transformer is the lack of interpretability. Why the Molecular Transformer predicts one reaction outcome over another, and which training set reactions it finds most similar when reaching a particular prediction, are both unclear. Quantitative interpretability is crucial to both model users and model developers.

For model users, interpretability is important because chemical reactions are highly contextual, with important anthropomorphic metadata that the model overlooks. For example, reactants, reagents and products are only a part of the reaction. The reaction conditions, the scale of a particular reaction (e.g. discovery chemistry or scale up), and scientific focus of the project (e.g. total synthesis, medicinal chemistry or methods development) are some of the context that a skilled chemist can employ to interpret and understand the reaction.

For model developers, physical organic chemistry principles explain chemical reactivity and selectivity. As such, probing whether rationales outputted by the Molecular Transformer are congruent with physics allows developers to interrogate whether the Molecular Transformer is getting the correct prediction for the right reasons, and design model improvements based on those insights.

In this paper, we develop a suite of methods that quantitatively interprets the Molecular Transformer by attributing predictions to the input chemical structure and the training data. We illustrate our two-prong approach via a series of sentinel examples, showing how we uncovered what the model is learning, what it finds difficult, and explains its failure modes. Our method discovers hidden biases in the training data that hinder generalization performance and masks model shortcomings, which we resolved by introducing a new unbiased train/test split.

## Results

### Quantitative interpretation

There are three key factors determining the prediction of a machine learning model: the architecture, the training data and the input. Neural network models are often considered as black-boxes because of the complex ways these three factors interact to yield a prediction.

To interpret model prediction, we first need to define what interpretability means. We suggest interpretability is the ability to discover associations and counterfactuals between input and output, and the ability to query evidence in the data supporting a certain outcome. Our approach follows the accepted scientific process: a scientific theory usually identifies factors that are related to a certain outcome and conversely how the absence of those factors is related to the absence of outcome. Furthermore, the investigator needs to show pieces of evidence that support the theory.

**Fig. 1:**
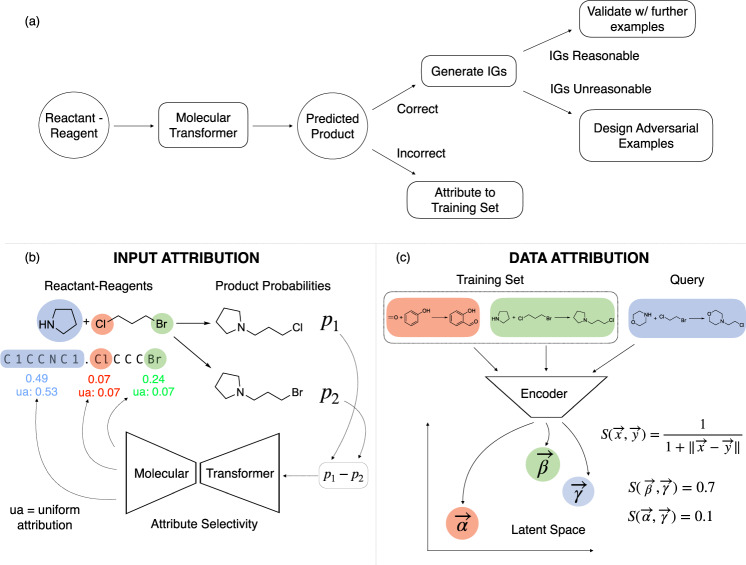
Schematic illustration of the attribution workflow. **a** Overview of our workflow to interpret the Molecular Transformer. **b** Schematic of how the predicted probability difference between two products are attributed back to the reactant-reagent string in order to interpret the model’s understanding of selectivity. The IG attributions below the reactant SMILES are compared to the uniformly distributed probability difference (ua) below. **c** Schematic of how the latent space encoding of reactant-reagent strings are used to infer the learnt similarity between query reactants and those from the training set.

We employ integrated gradients^16^ as a rigorous method for attributing the predicted probability difference of two plausible products of a selective chemical reaction to parts of the input. The attributions show how much each substructure is contributing to the predicted selectivity of the model. This is illustrated in Fig. 1b. The values of the attributions are compared to the value each subgroup would receive if the probability difference would be distributed evenly across the input. The parts of the structures getting higher integrated gradients (IGs) than the uniform attribution (ua) are considered important. For further description of our adaptation of IGs see Methods section.

Attributing the predictions of neural networks to most similar training data points is less widely researched. To achieve this goal, we developed a new method based on the latent space similarity of the reactions. We used the outputs of the Molecular Transformer encoder averaged over the tokens to achieve a fixed-length vector representation of the reactions. The most similar training reactions according to the model were then identified using the Euclidean distance of these latent space vectors. A schematic overview of our method is shown in Fig. 1c. Details can be found in the Methods section.

We validate our interpretations in two ways. The first is via falsification. If the integrated gradients attributions are chemically unreasonable, i.e. predictions are correct for the wrong reasons, we design adversarial examples that force the model into wrong predictions. The second is by identifying causes for the prediction in the training data. If a prediction is wrong, we interrogate whether a similarly incorrect entry is in the training data.

### Investigation of specific reaction classes

We investigate in detail three reaction classes that are commonly used in medicinal chemistry. Through these examples, we illustrate each of the three branches in Fig. 1a. We first examine the selective epoxidation of alkenes which is an example where the Molecular Transformer is producing the right prediction for the right reason. We then turn to the Diels–Alder reaction, which is a scaffold-building transformation widely used in synthesis. We show that the Molecular Transformer is not able to predict this reaction. Following the bottom branch of Fig. 1a, we investigate it using data attribution and find that the USPTO dataset contains very few instances of Diels–Alder reactions, likely explaining why the model is not able to predict the outcome correctly.

Finally, we consider the Friedel–Crafts acylation reactions of substituted benzenes. We show that the Molecular Transformer predicts the right product for the wrong reason and validate our interpretation using a number of adversarial examples. We also demonstrate with the help of an artificial dataset how this behaviour is the result of dataset bias.

In light of the identified pathologies, we re-examine the reported 90% accuracy of the Molecular Transformer and demonstrate that it is partly the result of scaffold bias in the dataset. We propose a new train/test split that is free from this bias, and show that the performance of the Molecular Transformer decreases. We also show that the same issue exists for graph models as well by retraining one of the best reported graph model and observing a similar drop in accuracy.

#### Epoxidation

The oxidation of alkenes to form epoxides is an important intermediate reaction in many synthesis plans^17^. The common oxidant in these reactions are peroxy compounds. The most widely used example of them is mCPBA, which is a versatile reagent appearing 2052 times in the USPTO dataset. This is in the high data regime where we would expect the model to do well due to the large number of different training examples available.

**Fig. 2:**
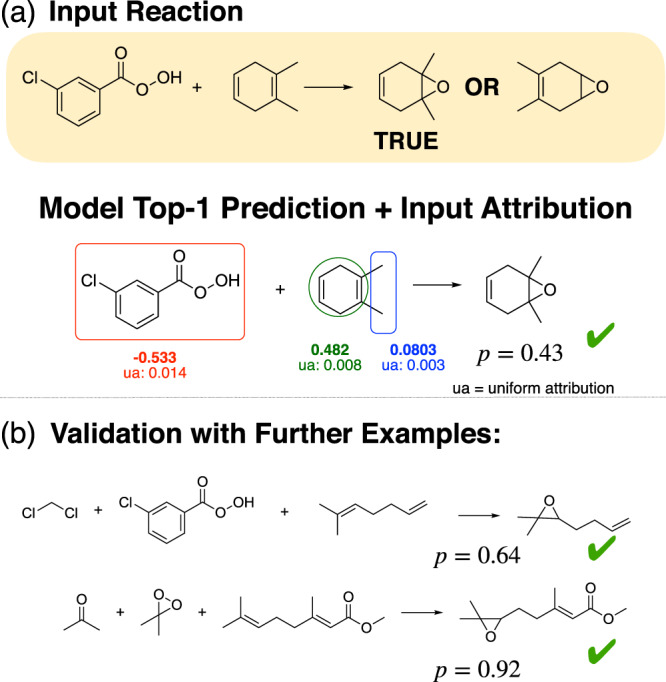
IG attributions highlighting correct reasoning. **a** The model correctly predicts the product of a typical epoxidation reaction, and shows significant positive attributions to the two methyl group that are responsible for the selectivity. **b** We validate the model’s knowledge on two unseen epoxidation reactions from chemical literature^39^.

Epoxidation reactions can be regioselective, with more substituted alkenes reacting faster because they are more electron-rich^17^. A typical example reaction showing this type of selectivity is shown in Fig. 2a.

The Molecular Transformer is able to predict the product with the correct selectivity, giving it a probability score of 0.43. The probability score of the alternative incorrect product was less only by 0.025. This is a case where the model predicts two similarly plausible outcomes, so IGs can help to judge whether or not a prediction can be trusted. Since the probability difference is close to 0, the sign of the attributions at different parts of the input is in itself interesting and contains information regarding the favoured outcome.

Figure 2a shows the IG attributions of the different parts of the input. In this case the positive attributions favour the correct product while the negative attributions favour the incorrect product. The IGs show that the two methyl substituents circled with blue are significantly contributing to the correctly predicted selectivity. The attributions on the other parts of the molecule are harder to interpret. This can be the result of the model being uncertain in the prediction leading to larger gradients along the path integral during the calculation of the attributions.

To validate the interpretation that the model has learnt this selectivity, we generated the Molecular Transformer predictions for two further examples from the literature as shown in Fig. 2b. The first example is very similar to the one examined in detail above and the model is consistently predicting the correct product. The second example is more challenging for the model for a number of reasons. First the reagent is not mCPBA but dimethyldioxirane which appears much less frequently, only 14 times in the training data, secondly both double bonds are substituted, and the difference is made by a more subtle chemistry, the ester group being electron withdrawing. The model is able to predict the correct outcome here as well confirming that the predictions are correct for the right reason.

#### Diels–Alder

**Fig. 3:**
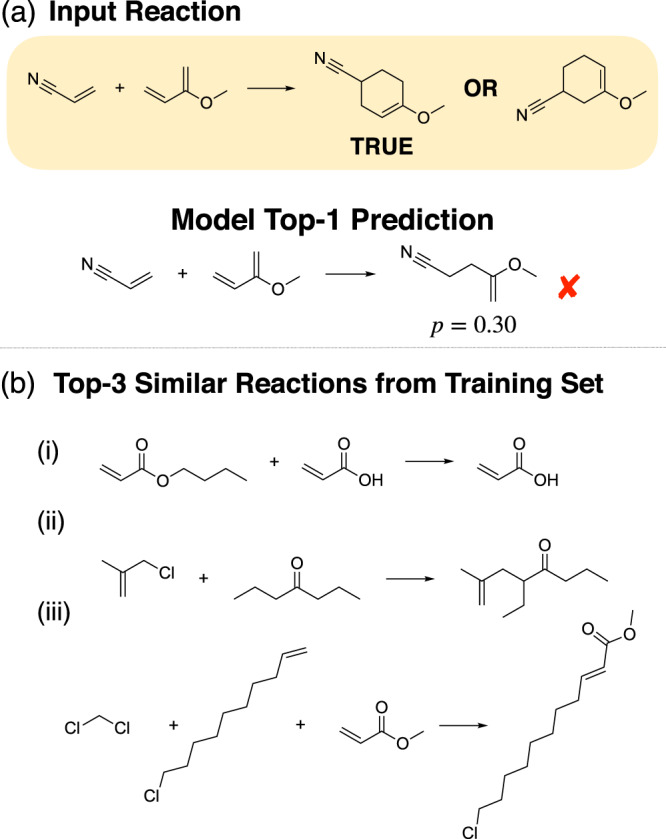
Data attribution explains erroneous prediction. **a** The model makes an obviously incorrect prediction on a typical example of a Diels–Alder reaction with challenging selectivity. **b** Attribution to the USPTO training data shows that the model either completely fails to recognize Diels–Alder reactions or that no Diels–Alder reaction is present in the dataset.

The Diels–Alder reaction transforms a conjugated diene and an alkene (called dienophile) to a six-membered ring with a double bond^17^. There are very few limitations on the character of the diene. It only has to be flexible enough to take up an s-cis conformation. The dienophile, on the other hand, should have carbon–carbon double bonds conjugated preferably with an electron withdrawing group. A typical example of a Diels–Alder reaction used as a test-case is shown in Fig. 3a.

The Molecular Transformer was unable to predict the regioselectivity of this reaction, and in fact the predicted product was clearly wrong with the actual possible products getting 0 probability scores. Since the prediction is obviously wrong, we followed the bottom branch of the workflow at Fig. 1a and generated the most similar training reactions to see what causes this erroneous prediction.

Figure 3b shows the Top-3 most similar reactions from the training set based on the model encoder output similarities. The most similar training reaction (i) is an erroneous reaction, whilst the second and third are carbon–carbon bond formations, but via Grubbs methathesis^18^ rather than cycloadditions. This means that the model has not learnt a good representation of Diels–Alder reactions in the latent space.

To investigate if the cause of this was a lack of training data, we devised a reaction template corresponding to the [4 + 2] cycloaddition and found that there were only seven reactions matching it in the entire USPTO database. This example illustrates how attribution to data can be useful for identifying erroneous predictions caused partly due to erroneous data and partly due to the scarcity of training examples.

#### Friedel–Crafts acylation

**Fig. 4:**
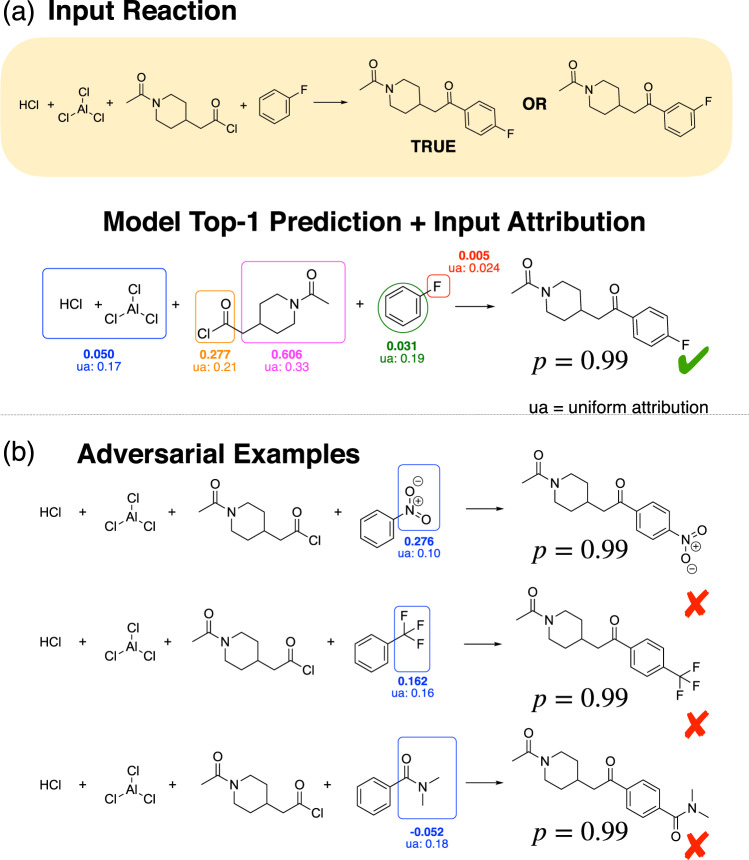
IG attributions revealing incorrect reasoning and guiding the design of adversarial examples. **a** The model correctly predicts the major para product of a typical Friedel–Crafts acylation, but low attribution is given to the para directing –F group. **b** The model is fooled into incorrectly predicting the para product when the –F is replaced by meta-directing groups. The low attributions given to the directing groups indicate that the model has not learnt their importance.

Friedel–Crafts acylation reactions are an example of electrophilic aromatic substitution^19^. In these reactions a hydrogen on an aromatic ring is substituted by an acyl group. In the case of a benzene ring with a single substituent, there are three different hydrogen positions where this substitution can happen. The electronic and steric character of the substituent on the ring determine the selectivity of these reactions. An example of a selective Friedel–Crafts reaction is shown in Fig. 4a where according to the patent the para product is formed with a yield of 90%^20^. In this reaction that acyl group is primarily substituting the hydrogen in the para position compared to the –F substituent. The transformation is correctly predicted by the Molecular Transformer.

The IG attributions indicate that the importance of the fluorine (–F) for this reaction is completely neglected by the model. A much larger attribution is given to the reagent suggesting that the model attributes this selectivity to the reagent rather than the true directing group. Guided by the attributions, we replaced the fluorine by a number of typical meta directing groups to create adversarial examples. We observe that the model (wrongly) predicts the para product. In this case, negative attributions favour the meta product and positive attributions the para product. We do not find any correlation between the attribution values and the directing effect of the substituent. From this, we can conclude that the model has not learnt the selectivity in the case of Friedel–Crafts acylation reactions on substituted benzene rings.

**Fig. 5:**
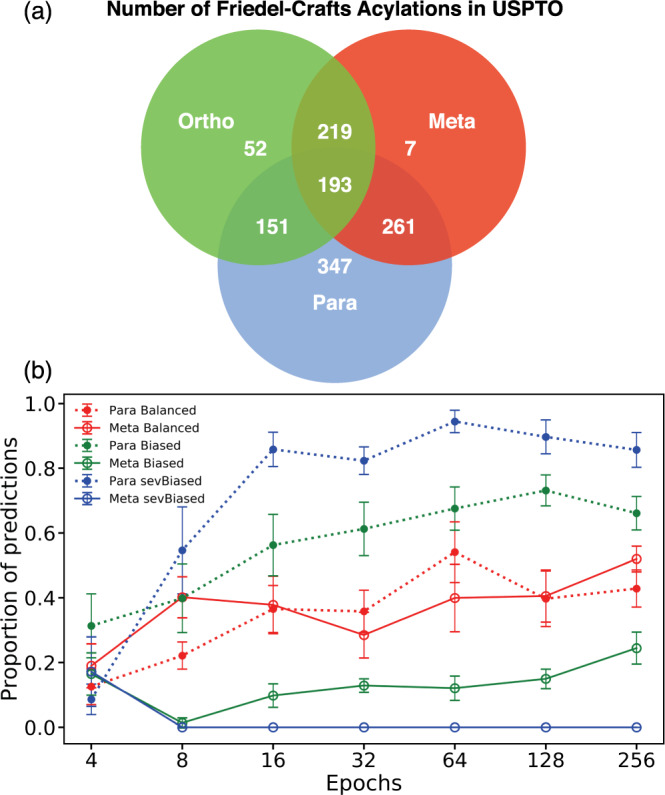
The effect of biased training data on the predictions of the Molecular Transformer. **a** The number of para Friedel–Crafts acylation reactions far outweigh those of meta or ortho reactions. **b** Dataset bias is reflected in the model predictions. The figure shows the proportion of para (solid line) and meta (dashed line) predictions on a balanced test set as a function of the number of training epochs for different biased training sets. The error bars shown indicate the standard deviation in the results from training an ensemble of 10 randomly initialized models. The proportion of meta and para predictions does not always add up to 1, because it takes a number of iterations for the model to learn the SMILES syntax and we discount invalid predictions.

Interestingly in our third example, the attribution on the meta directing group is negative, meaning that according to the model the amide group (correctly) favours the formation of the meta product. This agrees with chemical principles, but the model is nonetheless still predicting the para to be the major product. We hypothesize that this might be due to biases in the training data—Fig. 5a shows that there are many more para substitution reactions than meta in the training dataset; overlaps in the Venn diagram denotes cases where the benzene ring has more than 1 substituent. This could result in the model being biased towards predicting para substitutions even in the presence of meta directing groups, as the model can achieve very high (98%) accuracy on the training set by always predicting the para product.

### Revealing the effect of bias through artificial dataset

To investigate how imbalance in the training data affects the test set performance, we construct three artificial training sets using reaction templates for meta and para Friedel–Crafts substitutions.

The first training set is balanced, containing the same number of para and meta products. The second dataset contains 10% meta and 90% para products, whilst the third dataset has ca 1% meta and 99% para products. This last ratio is closest to the ratios of the USPTO dataset. The test set for all models contains an equal number of meta and para reactions.

Figure 5b reveals that the Molecular Transformer is highly susceptible to learning dataset bias. When the model is trained on the balanced dataset, it rapidly converges to predicting equal amounts of para and meta substitution reactions, confirming that the bias is not caused by neural network architecture limitations. The model trained on the biased dataset containing only 10% meta reactions in the training set is not able to get rid of the bias fully, but with longer training it is mitigated. For the highly biased training set the model is not able to learn to predict any meta products.

This numerical experiment confirms that the Molecular Transformer is guilty of the Clever Hans effect—it appears to know chemical reactivity only because it learns hidden bias in the dataset. This is analogous to the bias observed in neural machine translation, where a pronoun indicates the gender of a word, but the model disregards it when making the translation due to the presence of gender stereotypes in the training data^21^.

### Uncovering scaffold bias

Our case study of the Friedel–Crafts acylation reveals the sensitivity of the Molecular Transformer to dataset bias. We turn to examine another source of bias—compound series bias, or scaffold bias^22^. This is the phenomena where very similar molecules appear in both the training and the test set. This leads to ML models achieving high accuracy on the held-out set, which does not necessarily correlate with the true generalization performance of the models. This is particularly acute for drug discovery datasets as medicinal chemists typically design molecular ‘series’ by adding various functional groups to a central chemical ‘scaffold’. In chemical reaction datasets, scaffold bias manifest itself as similar molecules undergoing very similar transformations.

**Fig. 6:**
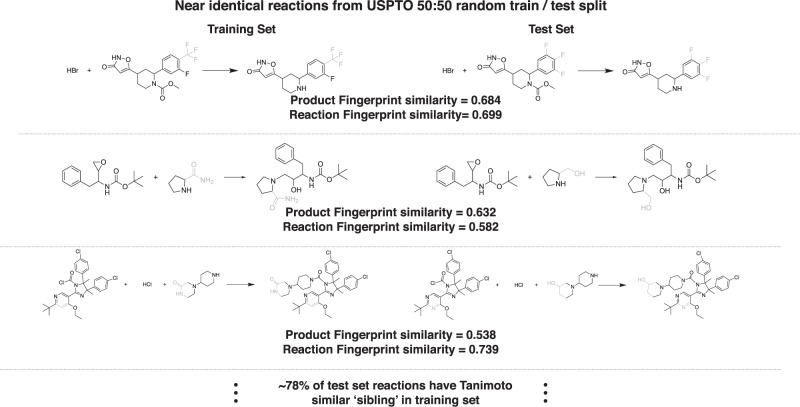
Near-identical training and testing reactions during random splitting of the dataset. Randomly splitting USPTO results in a large number of near-identical reactions shared between train/test sets. 78% of reactions in the test set have products that are within Tanimoto similarity 0.5 of a product in the training set following a 50:50 random split. By eye, it can be seen that many reactions with similar products (differences are highlighted by shading) have similar reagents and follow near-identical reaction mechanisms. This intuition is confirmed by the similarly high similarity of the reaction difference fingerprints from the reactions. The equivalent proportions are 93% and 57% for Tanimoto similarity >0.4 and >0.6, respectively.

To gain further insight into this phenomenon, we apply a 50:50 random train/test split to the full USPTO dataset and inspect reactions from one set that have structurally similar products to those from the other set. We define the ’structural similarity’ of two molecules by calculating the Tanimoto similarity *σ* between the Morgan fingerprints of the respective molecules^23^. Figure 6 reveals that many training and test set reactions are remarkably similar as measured by both *σ* as well as the Tanimoto similarity of the reaction difference fingerprints of the reaction^24^.

We find that 57–93% of reactions from the test set contain a structurally similar product to a reaction from the training set. This would not be problematic if the data points involved different reactants and reagents reacting via different mechanisms to form the same product. However, this is not the case—reactions with similar products often also share reactants and undergo similar chemical changes. This means that using a random train/test split to assess the performance of reaction prediction models could be a misleading indicator of their ability to generalize. Indeed, this reconciles the seeming contradiction between the reported 90% top-1 accuracy of the Molecular Transformer and our findings above regarding the model’s fragility to reactions involving chemical selectivity.

To account for this drastic scaffold bias, we propose that datasets for training machine learning reaction prediction models should be split by the Tanimoto similarity of the reaction products. In other words, it should be ensured that no reactions in the test set have a product that is within Tanimoto similarity *σ* of any product from a training set reaction. We implement this by first conducting a random split of the dataset, and then transferring all reactions that fall foul of the Tanimoto similarity criteria from the test set to the training set—the proportion of the initial random split is adjusted until the desired final train/test ratio is obtained.

The intent of such a dataset split is to remove structural bias, but we must also make sure that the distribution of different reaction types in the train and test sets is still similar. This is important because we would like the test set score to reflect how well the model learnt the chemistry contained in the training set, and we are less interested in extrapolation to unseen reaction types. To characterize the new Tanimoto-split dataset, we used open-source template extraction code^25^, and we inspected the distribution of reaction types in both the training and test sets for the case of the random and Tanimoto-split datasets (see Supplementary Note 1). We find that the distribution of reaction templates is not substantially perturbed and for both random and Tanimoto-splits there are no reaction templates present in the test set that are not contained in the training set, i.e. all reaction types in the test set are ’seen’ by the model during training. In fact, Tanimoto-splitting increases the number of unique templates in the test set from ~3k to ~4.9k, suggesting that this splitting method can produce test sets that better represents the distribution of reaction types from the full dataset (~26k templates) compared to a random split. This is similar to an importance sampling scheme that helps sampling the tails of the distribution as well.

We apply this technique to USPTO with *σ* = 0.6 and *σ* = 0.4, and train the Molecular Transformer on these two datasets. We also train the WLDN5 model of Coley et. al.^25^, which is a widely-used graph-based machine learning reaction prediction model. This model explicitly represents molecules as graphs and considers reactions as series of graph edits instead of the Molecular Transformer’s text-based translation of SMILES strings.

Table 1 shows that the model performance of both the graph-based model and the Molecular Transformer significantly decrease upon debiasing the dataset, but Molecular Transformer continues to outperform WLDN5. These results show that scaffold bias affects both graph-based and sequence-based models, confirming that this bias is intrinsic to data and independent of model architecture. Importantly, this demonstrates that there is significant scope for improvement in the performance of reaction prediction, and that the 90% accuracy obtained for a randomly split dataset does not necessarily translate to real-life applications.

**Table 1:** Evaluation of reaction prediction models on different train-test splits.

Model	Top-1[%]	Top-3[%]	Top-5[%]
Original			
Molecular Transformer	**90.4%**	**94.6%**	**95.3%**
WLDN5	85.6%	92.8%	93.4%
Tanimoto similarity <0.6			
Molecular Transformer	**80.9%**	**88.2%**	**89.6%**
WLDN5	75.9%	86.2%	88.8%
Tanimoto similarity <0.4			
Molecular Transformer	**74.6%**	**82.9%**	**84.5%**
WLDN5	69.3%	80.9%	84.1%

The performance of the best ML models on various USPTO train/test splits are shown.

The accuracy of the best-performing model is highlighted in bold.

## Discussion

We developed a framework for quantitatively interpreting the predictions of Molecular Transformer, a state-of-the-art model for predicting the outcome of chemical reactions. We show that the model makes predictions based on patterns it recognizes and the statistics of the training data, but this does not necessarily coincide with the underlying chemical drivers of reactivity. This can result in erroneous predictions. Attributing the predicted probability to parts of the input allowed us to foresee these failure modes.

Through this interpretation framework, we discover that the model is susceptible to the Clever Hans effect, where the correct outcome is reached by learning bias. For example, the dataset contains orders of magnitude more para than meta electrophilic aromatic substitution reactions, and the Molecular Transformer frequently arrived at the correct test set prediction by simply memorising this fact. We believe that the inclusion of additional physical insight into models, as done in recent work incorporating explicit reaction mechanisms for reaction prediction^26^ and machine-learning regio-selectivity prediction^27^, could be an effective way of increasing model robustness against dataset bias. A possible way to accomplish this in Transformer models is via the augmentation of token embeddings with physical descriptors. Moreover, future efforts should focus on benchmarking other graph-based synthesis prediction tools such as the recent MEGAN architecture as well^28^.

We have also shown that incorrect predictions can be the result of erroneous training data points. This can be revealed using our method to attribute model predictions to training data. This method can also aid experimental chemists using the Molecular Transformer. The references corresponding to the most similar training reactions can be used to impute experimental conditions. This principle can be used in many scientific machine learning applications where the training data is generated via text-mining, which is known to lead to loss of important metadata, like reaction conditions.

Finally, we have shown that scaffold bias is a phenomena present in the published literature on reaction prediction. Many of the reactions in the test set have almost identical twins in the training set. This leads to an overestimation of the generalization performance of the models as reported in the literature. We have re-trained two of the leading models the Molecular Transformer and the graph-based WLDN5 model on our new Tanimoto-split dataset and found that the Top-1 accuracy of the models dropped significantly.

Our work highlights the importance of understanding and evaluating scientific machine learning models beyond looking at their accuracy on standard benchmark datasets. By rigorously applying interpretability techniques, we reveal how systematic weaknesses of the models can be uncovered, proving insights that facilitate the work of model developers. We believe further work into the use of input attribution and interpretability tools to critically analyse machine learning models for retrosynthesis, as well as other areas of computational science, is vital and necessary for continued refinement of predictive models.

## Methods

### Input attribution

To unpack the Molecular Transformer we decided to focus our efforts on reactions containing selective chemical transformations, which means that they have multiple plausible outcomes. These reactions are most fit for identifying if the model is making the predictions on true chemical basis because the underlying chemical causes are well established. Our general framework of interpreting chemical reactions is shown in Fig. 1a.

Once a suitable chemical reaction with two possible target molecules is chosen the Molecular Transformer probability scores of the products are generated. The difference in probability score between the true and the incorrect but plausible products is then attributed back to the reactant-reagent inputs.

Recently there were many methods developed and applied successfully for attributing the predictions of neural networks to parts of the input. Some of the most notable examples are LIME, SHAP, layer-wise relevance propagation (LRP) and integrated gradients^16,29–31^. These methods are designed to propagate back the output of the models in a fair way to determine the contribution (importance) of each of the input features to the prediction. There are several methods that have their roots in cooperative game theory and are proven to yield fair attributions as defined by the axioms of fairness^16^. For machine learning models where the gradients are not readily available, there are so-called Shapley-values and the closely related SHAP method^30^. For models such as the Transformer where the gradients are easy to evaluate the integrated gradients (IGs) method is a more natural choice^16^ though other methods such as LRP have also been applied successfully^32^. The IGs method has also been applied previously for interpreting language models in natural language processing applications and for designing adversarial examples in the context of question answering^33^. A graphical illustration of IGs is shown in Fig. 1b. Our approach builds on the work of McCloskey et  al. ^34^, who used IGs to understand binding prediction by graph neural networks on artificial datasets. We extend the method to Transformer architectures, and use it in the context of reaction predictions on real experimental data.

IGs are calculated by evaluating the path integral of the gradient of the output with respect to the input along a straight line path in the input space from a non-informative baseline to the input of interest.

Given a neural network denoted by the function $F:{{\mathbb{R}}}^{n}\to [0,1]$, the input $x\in {{\mathbb{R}}}^{n}$ and the baseline input $x^{\prime} \in {{\mathbb{R}}}^{n}$ the IG attribution of feature *i* is given by

1 $${{\rm{IG}}}_{i}(x)=({x}_{i}-x^{\prime})\int_{\alpha =0}^{1}\frac{\partial F(x^{\prime} +\alpha (x-x^{\prime}))}{\partial {x}_{i}}d\alpha$$

In the case of the Molecular Transformer *x* is the *N* × 256 dimensional embedding of the input SMILES string of length *N* and $x^{\prime}$ is the embedding of the ‘.’ token taken *N* times. This token is used in the SMILES language to separate different molecules and hence on its own bears no chemical information making it an ideal baseline choice. To obtain the total contribution of each of the input tokens the attributions are summed along the 256 dimensional embedding vectors.

Finally to make the attributions easier to interpret, we devised a few simple rules to map the token level attributions to chemically meaningful substructures. Reagents like sulphuric acid or meta-Chloroperoxybenzoic acid (mCPBA) are fed into the model by their full SMILES strings but in reality they act as single units as far as the reaction is concerned. Their attributions are more meaningful to look at as a whole rather than token by token. A related problem is with the attributions corresponding to special characters in SMILES like numbers or parentheses. To resolve this, we consider rings as single units and their attribution is calculated by summing over the ring atoms and numbers. This way the information about the relative positions of the ring substituents will also be included in the attribution of this part of the structure. Branches are also considered as single units and their attribution is the sum over their atoms and the parentheses specifying them.

For the attributions to be meaningful it is important to look at reactions where there are two possible products that have non-zero probability scores according to the model. This is crucial since for the prediction of a single product every token of the reactant is important, since missing a remote carbon would also result in a wrong prediction. By looking at the probability difference of two plausible products this effect can be eliminated and the attributions highlight the groups driving the chemical selectivity (according to the model). In particular, canonical SMILES for both products should be used to ensure the probability scores are non-negligible.

Finally, to determine if a particular group is important according to the model, we compare its attribution to the attribution that would fall onto it, if the probability difference was distributed evenly across the input tokens. Substructures that get substantially higher attribution than uniform are most important for the model when it favours one product over the other.

### Training data attribution

Attributing the predictions of neural networks to training data can serve as a tool for explaining predictions as well as gaining understanding of the models inner workings^35^. In cases when a model predicts something very unexpected to humans attributions to parts of the input can be difficult to make sense of. Sometimes it can be much more illustrative to see a couple of example inputs that the model finds similar. Usually seeing a number of similar examples can help humans identify patterns that may serve as the basis of the model’s prediction. This can either result in the discovery of new trends or laws in the scientific domain or it can reveal biases that the model has learnt. In the latter case this information can be used to improve the model or the dataset.

To create a successful method for attribution to data the most crucial element is the careful design of a similarity measure. The similarity should be defined such that it measures how similar two input data points are according to the model. For different neural network architectures different choices of similarity measures can be appropriate. In the case of feed-forward or convolutional architectures a natural choice is to define a fingerprint vector for each data point that consists of the neural networks layer outputs (activations) concatenated together. This similarity measure has been shown to be useful for judging the reliability of toxicity models predictions by comparing molecules not in the training set^36^. In the case of the Molecular Transformer, which has an encoder-decoder architecture the output of the encoder layers can be used as a basis for comparing data points. Since the encoder hidden states have a non-fixed length we take the average of them across the input tokens to obtain a fixed-length 256 dimensional vector representation for each of the reactions. Averaging is expected to work because of the relatively large dimensionality of the latent space. The size of the vocabulary of the USPTO dataset is 288 so there are almost as many orthogonal directions in the latent space as there are possible different input tokens. This is expected to lead to minimal loss of information upon averaging. For each reaction in the training set the 256 dimensional hidden state vector is generated and the matrix of training set reaction hidden states is saved as a binary. When a new example input is given to the model, it is passed through the Transformer encoder and the average hidden state vector of it is calculated. A schematic diagram depicting the method is shown in Fig. 1b. The similarity score of the input reaction vector **u** to a training set vector **v** is calculated by

2 $${\rm{score}}({\bf{u}},{\bf{v}})=\frac{1}{1+\parallel {\bf{u}}-{\bf{v}}\parallel }$$

The top-*n* most similar reactions from the training set are returned.

### Training data

The training data used in this study was obtained by the text mining work of Lowe^13^, where organic reactions were extracted from US patents filed between 1976 and 2016. The dataset was filtered by Jin et al.^14^ to remove duplicates and some of the erroneous reactions, which resulted in a set of ca 480,000 organic reactions. This dataset though much cleaner, it still contained a large number of erroneous reactions whose sole product were halogen ions, nitric, sulphuric or phosphoric acids, etc. We found that if these reactions are present in the training set the Molecular Transformer is learning them resulting in catastrophic overfitting and unchemical predictions in some cases. To eliminate this effect, we deleted a further ca 8000 reactions to obtain a dataset of 471,791 reactions. We used 377,419 for training, 23,589 for validation and 70,765 as a hold-out test set. The training set was augmented by an equal number of random equivalent SMILES strings following the protocol of Schwaller et al.^11^. We trained a Molecular Transformer model as described in the original paper and were able to achieve 88.8% Top-1 accuracy on the test set, similarly to the original paper. This model was used throughout the interpretability experiments.

An important aspect of the training data is that since it was extracted from patented reactions it naturally contains a number of biases. Firstly there are no negative results included meaning that any combination of reactants and reagents in the dataset leads to a well-defined product. This is in contrast to reality where often there is no reaction, or the product is a mixture of many different compounds. This bias will always be reflected in the machine learning models predictions. A further bias stems from the distribution of reaction types in the dataset. Most of the patented reactions come from the medicinal chemistry community leading to reactions popular amongst medicinal chemists being over-represented. This bias can be useful since the model learns the kind of reactions medicinal chemists like using^37^ but it also hinders generalization because popular reactions are not necessarily better as has recently been shown in the case of inorganic chemical reactions^38^.

### Generation of the artificial dataset

To investigate how bias in the training data affects the Molecular Transformer, we generated an artificial dataset of electrophilic aromatic substitution reactions using SMARTS templates. Each reaction consists of a benzene ring singly substituted with a directing group reacting with an acyl chloride to form either a para- or meta- acylated product.

Ten benzyl compounds with para directing groups (fluorobenzene, chlorobenzene, isopropylbenzene, tert-butylbenzene, N-phenylacetamide, N-phenylpropionamide, phenol, ethoxybenzene, isopropoxybenzene, sec-butylbenzene) and ten benzyl compounds with meta directing groups (N,N,N-trimethylbenzenaminium, (trifluoromethyl)benzene, benzaldehyde, acetophenone, methyl benzoate, ethyl benzoate, benzonitrile, nitrobenzene, methyl benzenesulfonate, ethyl benzenesulfonate) were used. The –R groups for the acyl chlorides were generated by enumerating straight carbon chains of length 2–8 with 0–1 C=C double bonds also using SMARTS templates. Acyl chlorides were obtained by placing an acyl chloride group onto a random sp3 carbon on each of the -R groups. The acyl chlorides are enumerated with the benzyl compounds to generate valid chemical reactions.

We vary the proportion of para:meta reactions in the training dataset and observe how the Molecular Transformer performs on a test set with an 1:1 proportion of para:meta reactions. We construct a ‘Balanced’ dataset which has a 1:1 ratio of para:meta reactions (3100:3100) by enumerating all acyl chlorides with all benzyl compounds. We also create a ‘Biased’ dataset which has a 9:1 para:meta ratio (2790:310) by performing a 10:1 random split on the acyl chlorides so that less meta reactions are present. Finally we generate a ‘Severely Biased’ dataset with 100:1 para:meta ratio (3000:30), which is closest to the observed ratio in USPTO, by performing a 33:1 random split on the acyl chlorides and also only keeping three meta-directing benzyl compounds (benzaldehyde, (trifluoromethyl)benzene, and nitrobenzene).

The test set has an equal proportion of para and meta reactions generated using the three meta directing benzyl compounds from the ‘Severely Biased’ training set and three para directing ones (fluorobenzene, N-phenylpropionamide, and ethoxybenzene), together with –R groups from enumerating straight carbon chains of length 9–10 with no double bonds. This resulted in a test set with 177 para and 177 meta reactions.

### Tanimoto-splitting USPTO

Morgan fingerprints of radius 3 with 1024 bits were used to featurize the reaction products from USPTO. For the *σ* = 0.6 splitting, the initial dataset was randomly split 70%:30% and the ratio after Tanimoto splitting was 89.1%:10.9%. For the *σ* = 0.4 splitting, the initial dataset was randomly split 30%:70% and the ratio after Tanimoto splitting was 91.7%:8.3%.

## Supplementary information

Supplementary Information

## Data Availability

The USPTO dataset used to train the machine learning models is publicly available^13,14^, and Tanimoto similarity-based train/test splits of USPTO can found in the GitHub repo MTExplainer.

## Code availability

All code used for implementing the attribution tools for the Molecular Transformer, generating the artificial Friedel–Crafts dataset, and Tanimoto-splitting USPTO can be accessed in the GitHub repo MTExplainer. The transformer implementation was built on top of the OpenNMT-py package.
