# Supplementary material for: Quantitative interpretation explains machine learning models for chemical reaction prediction and uncovers bias
Source: Nat Commun. 2021 Mar 16;12:1695. doi: 10.1038/s41467-021-21895-w (PMC7966799; doi:10.1038/s41467-021-21895-w)
Supplement: Supplementary file 1 — Supplementary Information [file 41467_2021_21895_MOESM1_ESM.pdf]

Quantitative interpretation explains machine learning models for chemical reaction prediction and uncovers bias

Dávid Péter Kovács*[1], William McCorkindale*[1], and Alpha A. Lee[1†]

[1]Cavendish Laboratory, University of Cambridge, Cambridge CB3 0HE, United Kingdom

* Equal Contribution

†Correspondence Email Address: aal44@cam.ac.uk

Supplementary Information

Supplementary Note 1: Template Analysis of Distribution of Reaction Types from Tanimoto-Splitting

In order to inspect how the distribution of reaction types changes when using fingerprint similarity-based splitting, open-source template extraction code[1] was applied on the training, validation, and test sets from different dataset splitting methods. Reaction SMARTS describing bond changes of radius 1 were used to classify reactions to particular templates.

The frequency of occurrence for each reaction template is divided by the size of the training/validation/test set to obtain the fractional occurrence of the template, which is plotted in decreasing order of frequency in the training set. For rare templates (ie low frequency reaction types) floating point errors are encountered; however these do not affect the qualtitative arguments made.

These graphs are plotted for random split as well as Tanimoto similarity-based splitting using the Morgan fingerprint of the reaction product as well as reaction difference fingerprints at two different threshold values. These graphs show that distribution of templates in the test set closely follows that of the training set in all cases. As increasingly strict fingerprint similarity-based splitting is applied, the fractional occurrence of rare templates deviates more and more from that of the training set.

---

[1] https://github.com/connorcoley/rexgen_direct

Regarding the choice of fingerprint for similarity-based splitting, both simply using the fingerprint of the reaction product as well as the fingerprint difference between the product and reactants leads to qualitatively similar changes in the reaction template distribution. However, we have concerns that noise present in the data-mining of reactants and reagents (presence/absence of salt/catalysts/solvents etc) could cause unintentional effects on similarity calculation using reaction fingerprints and lead to additional hidden biases within the split. Together with the relative interpretability of the product fingerprint, we believe it is most practical to the community to simply use the fingerprint similarity of the reaction products for splitting datasets.

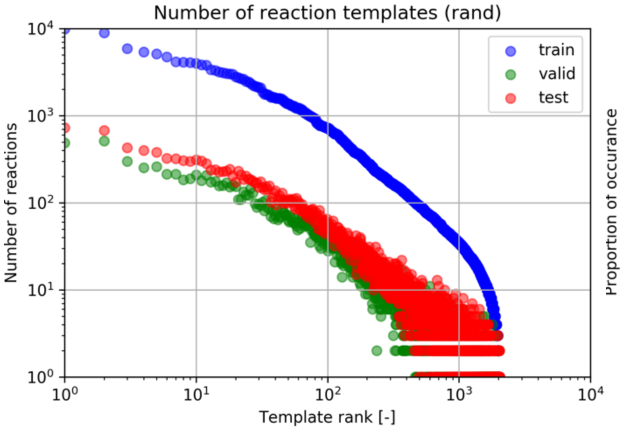

Supplementary Figure 1. The absolute occurrence (left) and fractional occurrence (right) of reaction templates in train/valid/test sets of USPTO from a random split. The distribution of test set reactions closely resembles that of the validation set.

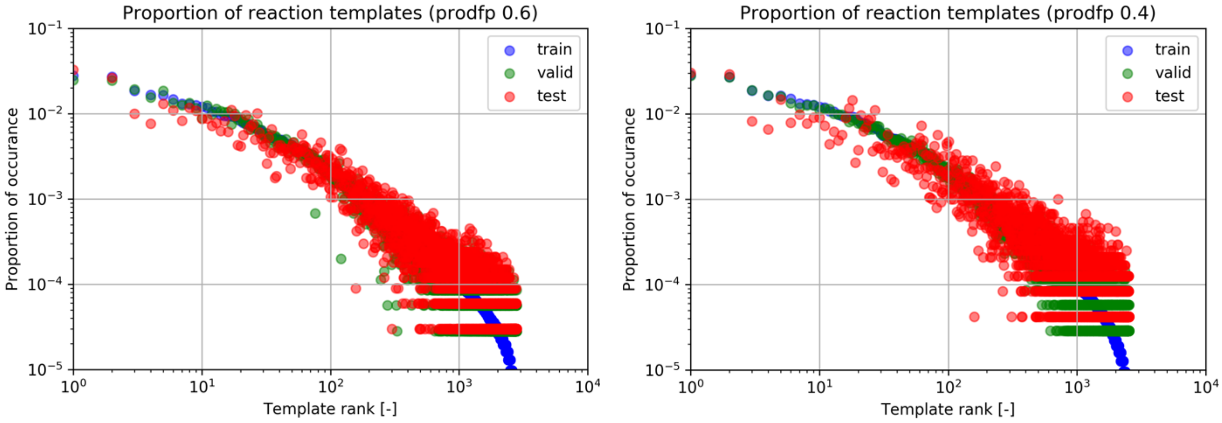

Supplementary Figure 2. The fractional occurrence of reaction templates in train/valid/test sets of USPTO from two different Tanimoto splits using the Morgan fingerprint of the reaction product. As the Tanimoto similarity threshold value is tightened from 0.6 (left) to 0.4 (right), the deviation in frequency of the test set reactions from the training set increases.

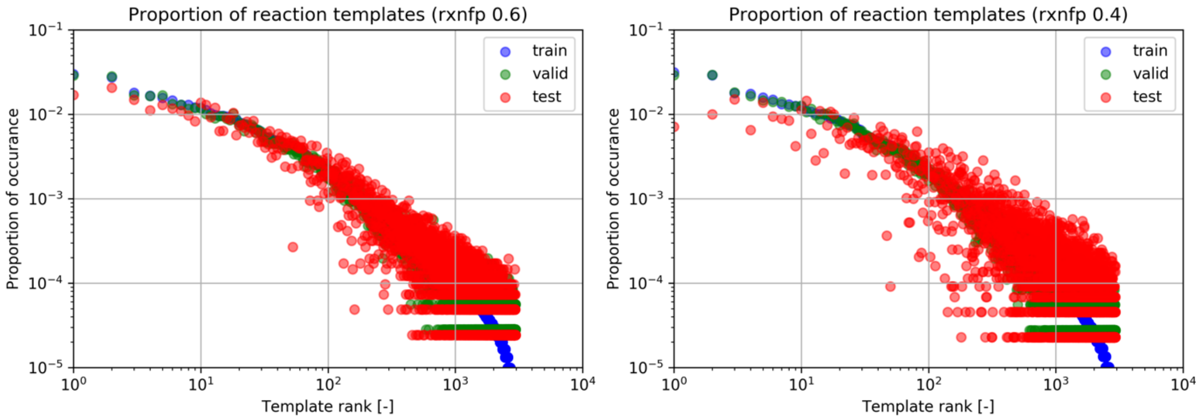

Supplementary Figure 3. The fractional occurrence of reaction templates in train/valid/test sets of USPTO from two different Tanimoto splits using the reaction difference fingerprint of the reaction. As the Tanimoto similarity threshold value is tightened from 0.6 (left) to 0.4 (right), the deviation in frequency of the test set reactions from the training set increases even more so compared with just the Morgan fingerprint of the reaction product, in particular for non-rare templates.
